# Aldehyde Dehydrogenase Mutation Exacerbated High-Fat-Diet-Induced Nonalcoholic Fatty Liver Disease with Gut Microbiota Remodeling in Male Mice

**DOI:** 10.3390/biology10080737

**Published:** 2021-08-01

**Authors:** Sien-Sing Yang, Yi-Hsun Chen, Jui-Ting Hu, Ching-Feng Chiu, Shao-Wen Hung, Yi-Chih Chang, Chien-Chao Chiu, Hsiao-Li Chuang

**Affiliations:** 1 Liver Center, Cathay General Hospital Medical Center, Taipei 106, Taiwan; yangsien@hotmail.com (S.-S.Y.); jaab@cgh.org.tw (J.-T.H.); 2 Department of Internal Medicine, College of Medicine, National Taiwan University, Taipei 100, Taiwan; u9423201@gmail.com; 3 Graduate Institute of Metabolism and Obesity Sciences, Taipei Medical University, Taipei 110, Taiwan; chiucf@tmu.edu.tw; 4 Division of Animal Industry, Animal Technology Research Center, Agricultural Technology Research Institute, Miaoli 350, Taiwan; 1032169@mail.atri.org.tw (S.-W.H.); chiu2295@yahoo.com.tw (C.-C.C.); 5 Department of Medical Laboratory Science and Biotechnology, Asia University, Taichung 413, Taiwan; yichih@asia.edu.tw; 6 National Laboratory Animal Center, National Applied Research Laboratories, Taipei 115, Taiwan

**Keywords:** aldehyde dehydrogenase 2, metabolic syndrome, oral glucose tolerance, gut microbiota

## Abstract

**Simple Summary:**

ALDH2, mitochondrial aldehyde dehydrogenase 2, is a critical enzyme involved in ethanol clearance in acetaldehyde metabolism. The prevalence of the ALDH2*2 variant is 45% in the Taiwanese population. ALDH2 reportedly has protective properties on myocardial damage, stroke, and diabetic retina damage. However, the effects of ALDH2 in modulation of metabolic syndromes remain unclear. The study evaluated the roles of ALDH2 in a high-fat-diet-induced metabolic syndrome in mice. We explored the effects of ALDH2 gene on NAFLD and potential association with gut microbiota.

**Abstract:**

Mitochondrial aldehyde dehydrogenase 2 (ALDH2) is a critical enzyme involved in ethanol clearance in acetaldehyde metabolism and plays a key role in protecting the liver. The ALDH2*2 mutation causes a significant decrease in acetaldehyde scavenging capacity, leading to the accumulation of acetaldehyde after consuming alcohol. The prevalence of the ALDH2*2 variant is in 45% of Taiwanese individuals. ALDH2 reportedly has protective properties on myocardial damage, stroke, and diabetic retina damage. However, the effects of ALDH2 in the modulation of metabolic syndromes remain unclear. This study evaluates the roles of ALDH2 in a high-fat-diet-induced metabolic syndrome in mice. Male (M) and female (F) wild-type (WT) and ALDH2 knock-in C57BL/6J mice (4–5 weeks old) were fed a high-fat diet for 16 weeks. Results showed that the body and white-adipose-tissue weights were significantly increased in ALDH2-M compared to those in the other groups. We observed markedly elevated serum levels of alanine transaminase and glucose. Oral glucose-tolerance test and homeostasis-model assessment of insulin resistance (HOMA-IR) values were significantly higher in ALDH2-M mice than those in WT-M mice, with no observable differences in female mice. Abundant steatosis and inflammatory cells were observed in ALDH2-M, with significantly decreased expression of hepatic genes IRS2, GLUT4, and PGC-1α compared to that in WT-M. ALDH2 gene mutation also affected the β-diversity of gut microbiota in ALDH2-M resulting in the decreased abundance of *Actinobacteria* and an increase in *Deferribacteres*. Our results suggest that potential changes in gut microbiota may be associated with the defective ALDH2 exacerbation of high-fat-diet-induced liver diseases in male mice. However, female mice were not affected, and sex hormones may be an important factor that requires further investigation.

## 1. Introduction

Aldehyde dehydrogenase 2 (ALDH2) detoxifies toxic aldehydes and plays a key role in protecting the liver [1]. The amount of ALDH2 in the liver is abundant. The ALDH2*2 allele variant is a single point mutation (G to A) in exon 12, resulting in a change from glutamine to lysine (E487K) and the inactivation and deficiency of ALDH2 enzyme activity in humans. ALDH2 activity is almost null in ALDH2* 2 homozygotes, and ALDH2*1/*2 heterozygous genotype has normal ALDH2 enzyme activity of 17–38% in humans [2]. The ALDH2*1/*2 mutation causes a significant decrease in acetaldehyde scavenging capacity, leading to the accumulation of acetaldehyde after consuming alcohol [3]. ALDH2*1/*2 is a common variant in about 40% of East Asians, with a prevalence ranging from 28% (Korea) to 45% (Taiwan) [4]. The ALDH2*1/*2 mutation is more susceptible to alcoholic liver disease [3]. Furthermore, Oniki et al. reported that the ALDH2*2 allele significantly influences the risk for NAFLD [5]. Hao and Zeng showed that ALDH2 rs671 GA and AA genotypes are factors associated with increased NAFLD among Chinese subjects [6]. These results support a role for alcohol-metabolizing enzymes in NAFLD pathology, and further studies are needed to clarify the effects of NAFLD in alcohol metabolism and how alcohol affects NAFLD pathogenesis.

NAFLD is characterized by hepatic steatosis without a history of excessive alcohol use [7]. However, alcoholic liver disease (ALD) with heavy ethanol consumption produces hepatic lesions, including early steatosis [8]. In the case of continued consumption of alcohol, fatty liver can progress to fibrosis and cirrhosis, which finally results in portal hypertension and liver failure [9]. Hepatic steatosis plays a major role in the histological diagnosis of NAFLD [7]. The interplay among genetic background, diet, and microbiota plays a crucial role in the complex pathogenesis of NAFLD. The pathogenesis of NASH is based on a three-hit hypothesis: steatosis represents the ‘first hit’, which then sensitizes the liver to injury, mediated by ‘second hits’ such as inflammatory cytokines, oxidative stress, and mitochondrial dysfunction, leading to steatohepatitis and fibrosis. In addition, oxidative stress reduces the ability of mature hepatocytes to proliferate. The impaired proliferation of hepatocyte progenitors represents the proposed ‘third hit’ in NAFLD pathogenesis [10]. Human and animal studies demonstrated a potential causal role of the gut microbiota in NAFLD. Mice received human NAFL microbiota and were subsequently fed a high-fructose, high-fat diet, leading them to gain more weight and have a higher number of liver triglycerides than that of mice that received healthy human microbiota [11]. The abundance of *Ruminococcaceae* and *Tannerellaceae* was increased, and the abundance of *Desulfovibrionaceae* and *Rikenellaceae* was decreased in the mice receiving human NAFL microbiota [11]. Fei and colleagues reported that liver inflammation was dependent upon endotoxin expression in HFD-fed NAFLD mice [12]. Animal studies demonstrated a potential causal role of gut microbiota in NAFLD [11,13]. *Proteobacteria* are enriched in NAFLD and NASH [14]. Rau et al. showed that the gut microbiota of NAFLD patients had reduced diversity compared with that of healthy individuals [15]. These studies showed that the gut microbiota plays a role in NAFLD development.

Obesity is a major risk factor in the development of insulin resistance (IR), which promotes the development of NAFLD [16]. ALDH2*1/*2 may increase the risk of noninsulin-dependent diabetes mellitus (NIDDM) [17]. The consumption of ethanol of more than 20 times per week may increase insulin resistance in humans with normal ALDH2 genotypes; however, insulin resistance is still observed in people with the ALDH2*1/2* genotype who consume much less ethanol [18]. Men also have more visceral and hepatic adipose tissue, whereas women have more peripheral or subcutaneous adipose tissue. These differences, and differences in sex hormones and adipokines, may contribute to a more insulin-sensitive environment in women than that in men. Volzke et al. reported that endogenous estrogens play a protective role in NASH, which may explain why the prevalence of NAFLD increases in females over 50 years of age [19]. Overall, gender may also affect the severity of IR and the pathogenesis of NAFLD.

Our study used ALDH2*1/*2 knock-in mice, which have a similar phenotype of elevated acetaldehyde levels after alcohol challenge, similar to the human ALDH2*1/*2 [20]. This mouse model mimics the human ALDH2*1/*2 point mutation better than ALDH2 knock-out mice do. The ALDH2*2 allele and gender may be associated with an increased risk for NAFLD; therefore, we explored the effects of ALDH2 and gender on NAFLD as a factor of changing gut microbiota, and the potential association with insulin resistance.

## 2. Materials and Methods

### 2.1. Animals

The specific-pathogen-free ALDH2*/1*2 genotype and wild-type male mice were gifts from Dr. Che-Hong Chen (Stanford University, California). An ALDH2*1/*2 knock-in mouse was developed by replacing the mouse wild-type ALDH2 allele with a mouse E487K mutant ALDH2 allele by homologous recombination. ALDH2*1/*2 knock-in mice differ only by a single amino acid within the ALDH2 gene compared with wild-type mice [20]. The mice were maintained in a specific-pathogen-free (SPF) animal room, and the same gender and genotype mice were kept in an individually ventilated cage with aspen chip bedding (Tapvei, Harjumma, Estonia), 2–3 mice/cage. Animals were maintained at room temperature (23 ± 2 °C), with 40–70% relative humidity, and a 12 h light/dark cycle.

### 2.2. Experimental Design

Male (M) and female (F) ALDH2*1*/2 genotype and wild-type mice (4–5 weeks old; grouped as WT-M, ALDH2-M, WT-F, and ALDH2-F) were maintained on a standard rodent diet, were given sterile water to drink ad libitum for an acclimation period of 1 week, and then fed a high-fat diet (HFD; *n* = 5–6, respectively). The diet was composed of 20%, 20%, and 60% calories from protein, carbohydrates, and fat, respectively (D12492, Research Diets Inc., New Brunswick, NJ). Mice were provided with water ad libitum during the 16 weeks of the study and weighed weekly. At the end of 16 weeks, mice were euthanized by asphyxiation with 95% CO2. Livers were extracted, fixed in 10% neutral buffered formalin for 24 h, and subjected to histopathological analysis. For the preparation of frozen sections, tissue samples were fixed with a tissue-embedding medium compound (Tissue-Tek O.C.T. Compound, Sakura Finetek, Torrance, CA, USA), and stored at −80 °C until sectioning with a cryostat. Liver-tissue samples for gene-expression analysis were stored in liquid nitrogen [13].

### 2.3. Oral Glucose-Tolerance Tests and Homeostasis-Model Assessment of Insulin Resistance

Oral glucose-tolerance tests (OGTT) were performed after 15 weeks on the designated diet. Oral glucose solution was administered to animals at 2 g/kg body weight by oral gavage, and tail-vein blood was collected at 0, 15, 30, 60, and 120 min after glucose administration. Blood-glucose concentrations were measured using Ascensia ELITE™ XL (Bayer AG, Zurich, Switzerland). Plasma insulin was measured at 120 min after glucose administration. Briefly, samples were centrifuged at 2500× *g* for 10 min at 4 °C, and blood insulin was quantified using a rat/mouse insulin ELISA kit (Millipore, St Charles, MO, USA), according to the manufacturer’s protocol. Homeostasis-model assessment of insulin resistance (HOMA-IR) was calculated as (fasting glucose level (mmol/L) × fasting insulin level (μU/mL))/22.5.

### 2.4. Measurement of Biochemical Parameters

Whole blood was collected by cardiac puncture and centrifuged at 2600× *g* for 10 min at 4 °C. Serum was immediately stored at −80 °C until the analysis of AST, ALT, GLU, TG, and T-CHO on a HITACHI 7080 automated analyzer (Hitachi, Tokyo, Japan).

### 2.5. Histopathological Examination

Liver-tissue samples were fixed in 10% neutral-buffered formalin for 1 day, dehydrated, embedded in paraffin, cut into 4 μm slices, and stained with hematoxylin and eosin (H&E) for histological examination. Steatosis severity was graded as 0 (<4%), 1 (5–33%), 2 (34–66%), or 3 (67–100%). Necrosis lesions, including inflammatory cells with necrosis hepatocytes, were graded as 0 (no foci per 200 × field), 1 (<2 foci per 200 × field), 2 (2–4 foci per 200 × field), and 3 (>4 foci per 200 × field) [13,21].

### 2.6. Oil Red O Staining

Fresh liver tissue was embedded in a Tissue-Tek 4583 OCT compound (Sakura Finetek, Torrance, CA, USA). The tissue was sectioned into 4 μm samples on a universal microtome cryostat (Leica CM3050S) (Leica Microsystems, Nussloch GmbH, Nußloch, Germany) and processed for the examination of fat accumulation by oil red O staining as previously described [13].

### 2.7. Measurements of Liver Triglycerides

Liver samples were weighed (~50 mg) and homogenized. The liver TG concentration of each sample was determined using a triglyceride colorimetric assay kit (Cayman, Ann Arbor, MI, USA).

### 2.8. Insulin-Signaling-Related Gene Expression

Total RNA was isolated from colon-tissue samples using an RNeasy minikit (Qiagen, Hilden, Germany). First-strand complementary DNA was synthesized using the Transcriptor First Strand cDNA synthesis kit (Roche Diagnostics GmbH, Basel, Switzerland). Quantitative real-time PCR was performed using TaqMan gene-expression assay (Universal Probe Library, Roche Diagnostics GmbH, Basel, Switzerland) in a LightCycler 1.5 (Roche Diagnostics GmbH). Cycle conditions were as follows: 95 °C for 10 min, followed by 40 cycles of 95 °C for 10 s, 60 °C for 25 s, and 40 °C for 30 s. β-Actin was used as an internal control, and nuclease-free water served as the negative control [13]. The sequences of primers used for analysis are listed in Table 1. The comparative Ct method was used to evaluate relative mRNA levels in the colon tissue samples.

### 2.9. Fecal Microbiota Analysis

Mouse fecal samples were collected, and DNA was extracted using the QIAamp^®^ DNA Stool Mini Kit (Qiagen Inc., Valencia, CA, USA). The V3-V4 regions of 16S rDNA were amplified to assess fecal-microbiota composition using Illumina HiSeq (Illumia, Inc., San Diego, CA, USA). The 18 raw sequence datasets were merged and processed with a uniform standard via QIIME2 pipeline version 2019.10. DADA2 was used to denoise the data and assess the sequence quality score (QS). The parameters for trimming and truncation settings were 17 and 260 for forward, and 21 and 260 for reverse. Samples with overall quality <30 were eliminated. Then, amplicon-sequence-variant (ASV) sequence data were annotated by the Silva 132 database, and a feature table was created. Then, 16S sequencing data were analyzed using the Vegan package in R software. Samples were rarefied to even sampling depths before computing within-sample compositional α-diversity and between-sample compositional β-diversity. Linear-discriminant-analysis effect size (LEfSe) was adopted for microbial comparison analysis (significance level of *p* < 005 and linear-discriminant-analysis (LDA) score > 2 were chosen to characterize the phenotype).

### 2.10. Statistical Analysis

Statistical analyses were performed using Graph-Pad Prism 6 software (GraphPad, Inc., La Jolla, CA, USA). Results are presented as mean ± SD. Statistical analyses were performed using the Kruskal–Wallis test followed by Dunn’s multiple-comparison post hoc test. Data with *p* < 0.05 were considered statistically significant. Two-way analysis of variance (ANOVA) was employed to compare multiple groups with Fisher’s least-significant-difference test. The significance of differences between histopathology scores was evaluated by the two-tailed Mann–Whitney U test. Data with *p* < 0.05 were regarded as statistically significant. 

## 3. Results

### 3.1. ALDH2-M Mice Have Higher Terminal Body and White Adipose Tissue Weights

We compared the body and white adipose tissue weights of WT and ALDH2 mice after 16 weeks of an HFD. ALDH2-M mice had significantly higher body and adipose tissue weights than those of WT-M mice (body weight *p* = 0.01; adipose tissue *p* = 0.004); there was no difference in the body weight and adipose tissue of WT-F and ALDH2-F groups (Table 2). There was no difference in animal food intake between the WT-M and ALDH2-M groups, or between the WT-F and ALDH2-F groups.

### 3.2. ALDH2 Male Mice Develop More Severe Hepatic Steatosis

**Figure 1:**
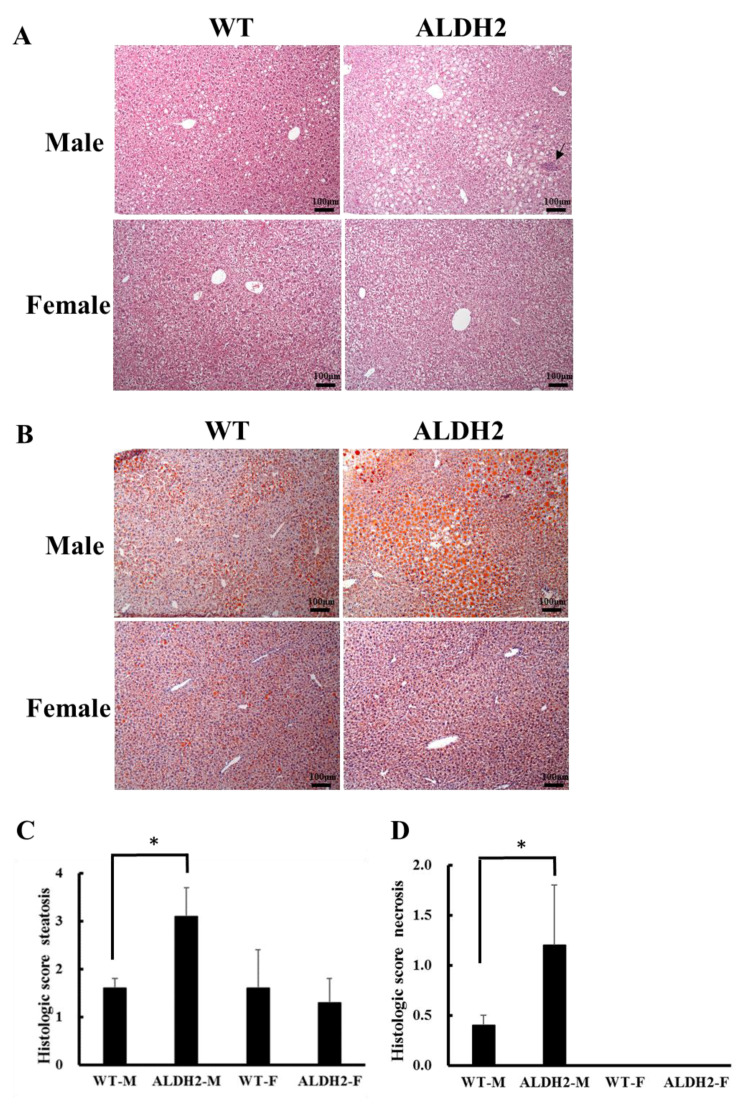
Histologic liver observation, fat drops, steatosis, and necrosis in wild-type and ALDH2 knock-in mice fed HFD. (**A**) H&E staining of liver sections; (**B**) oil red O staining of liver sections; (**C**) steatosis score of liver; (**D**) necrosis score of liver. H&E and oil red O staining (200×). Black arrow is necrosis. * *p* < 0.05 compared with WT-M.

The serum ALT level was significantly higher in the ALDH2-M mice than that in the WT-M mice (*p* = 0.0089); however, there was no marked difference in female mice. The serum AST level showed no difference (Table 2). As shown by liver histology, ALDH2-M exhibited more hepatic steatosis than WT-M (Figure 1A,B), and no or minimal hepatic oil droplets were seen in ALDH2-F and WT-F (Figure 1B). The average scores for steatosis were 3.1 ± 0.6, 1.6 ± 0.2, 1.3 ± 0.5, and 1.6 ± 0.8 in ALDH2-M, WT-M, ALDH2-F, and WT-F groups, respectively (Figure 1C). The mean necrosis score was 1.2 ± 0.6 for the ALDH2-M group, which was higher than 0.4 ± 0.1 for the WT-M group (*p* < 0.01) (Figure 1D). Consistent with H&E staining results on steatosis, oil red O-stained sections presented a larger degree of positive lipid droplets in ALDH2-M than that in other groups. ALDH2-M mice showed a marked accumulation of hepatic TGs in their liver tissue samples compared to WT-M. The levels of liver TGs in ALDH2-F were no different than those of WT-F (Table 2).

### 3.3. ALDH2 Gene Mutation Increases Insulin Resistance in ALDH2-M Mice

**Figure 2:**
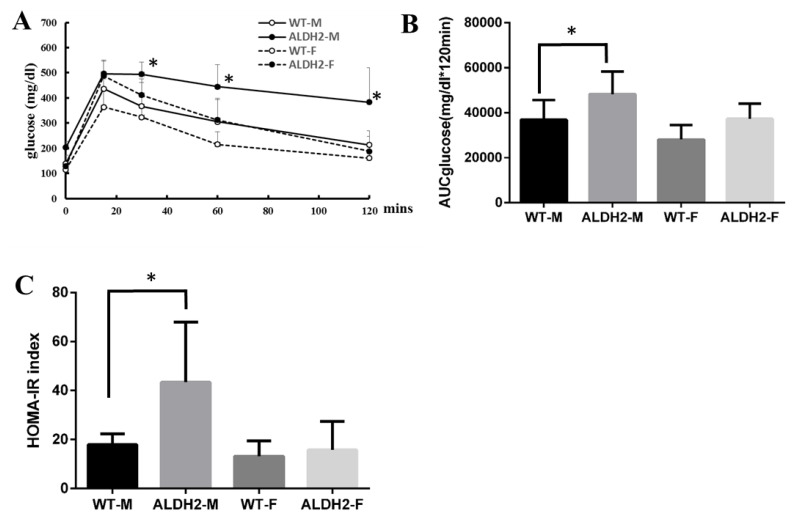
Oral glucose-tolerance test and HOMA-IR index. OGTT was performed at 6 h fasted (*n* = 5 and 6). (**A**) Glycemic values before (basal) and 15, 30, 60, and 120 min after glucose loading; (**B**) area underneath the curve (AUC); (**C**) homeostatic-model assessment for insulin resistance (HOMA-IR). Results are presented as mean ± SD. * *p* < 0.05 vs. WT-M.

To assess possible resistance to insulin, we examined glucose metabolism in mice after the HFD. Oral glucose-tolerance test (OGTT) results showed that blood glucose was significantly higher after 30, 60, and 120 min of glucose feeding in ALDH2-M mice than that in WT-M mice after glucose feeding for 30, 60, and 120 min (Figure 2A). Analysis of the area underneath the curve (AUC) showed that the ALDH2-M group had significantly higher blood-glucose levels than those of the WT-M group (Figure 2B). Furthermore, the HOMA-IR showed that ALDH2-M mice were less sensitive to insulin than WT-M mice. We did not observe any significant differences in glucose tolerance or insulin resistance in ALDH2-F mice compared with WT-F mice (Figure 2C).

### 3.4. Analysis of Gene Expression on Insulin Signaling Pathway

**Figure 3:**
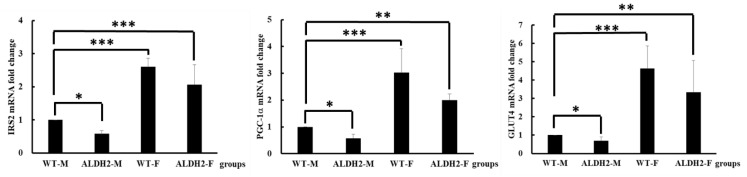
Gene-expression levels in liver tissue samples of wild-type and ALDH2 knock-in mice fed 60% high-fat diet. Data presented as mean ± standard deviation (SD). * *p* < 0.05, ** *p* < 0.01, *** *p* < 0.001 compared with WT-M.

GLUT4, PGC-1α, and IRS2 are critical for maintaining normal glucose homeostasis [22,23]. In our study, the gene expression of IRS2, GLUT4, and PGC-1α was significantly decreased in the ALDH2-M group compared with that in the WT-M group. The expression of these genes was significantly increased in the ALDH2-F and WT-F groups compared with that in the WT-M group (Figure 3).

### 3.5. ALDH2 Gene Mutation Affects Gut-Microbiota Composition and Diversity

**Figure 4:**
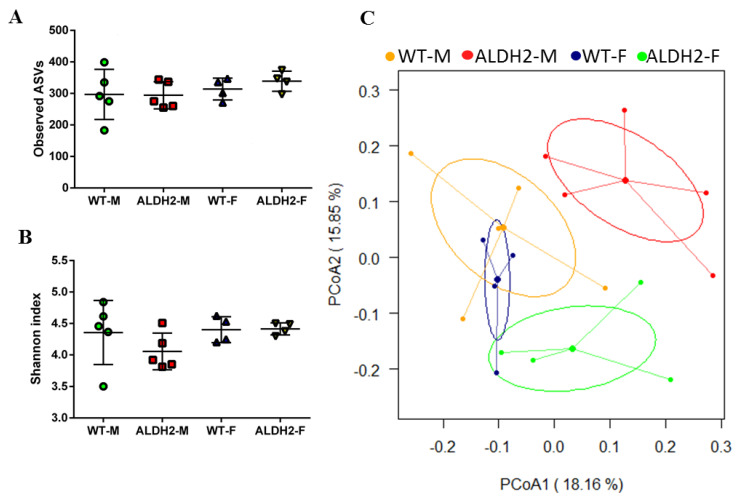
Microbiota composition and diversity in wild-type (WT) and ALDH2 mice. Microbiota composition in feces of HFD fed mice were analyzed using next generation sequencing (*n* = 4–5 for each group). (**A**) Observed amplicon sequence variants (ASVs); (**B**) Shannon index; (**C**) Principal coordinate analysis (PCoA). Significant differences between ALDH2*2 vs. WT indicated: *p* < 0.05.

**Figure 5:**
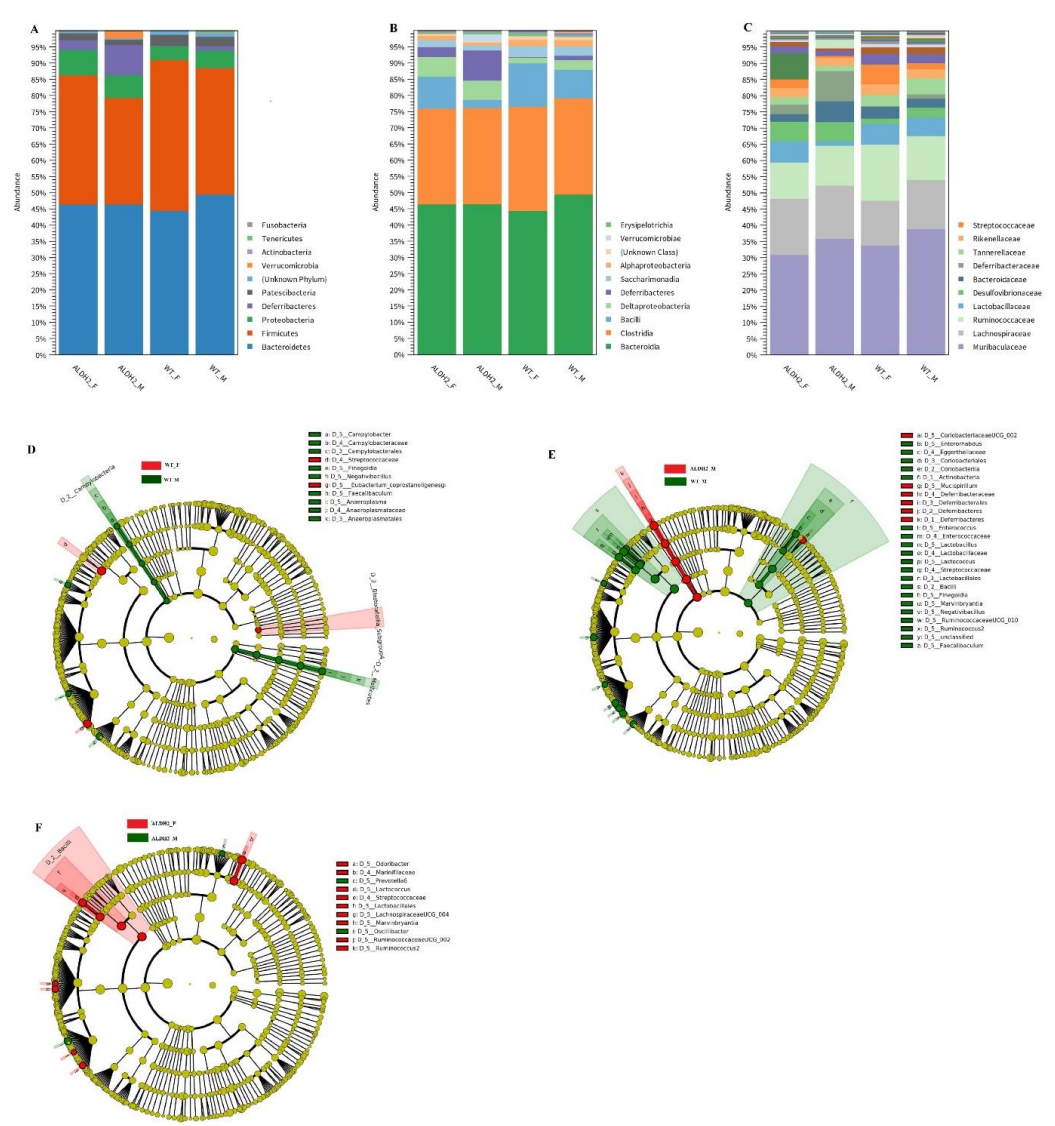
Gut-microbiota composition in wild-type (WT) and ALDH2 (**A**) phylum-level taxonomic distribution of microbial communities in fecal contents; (**B**) class-level taxonomic distribution of microbial communities in fecal contents; (**C**) order-level taxonomic distribution of microbial communities in fecal contents. Cladogram generated from LEfSe analysis showing most differentially abundant taxa enriched in microbiota between (**D**) WT-M and WT-F, (**E**) WT-M and ALDH2-M, and (**F**) ALHD2-M and ALDH2-F.

The gut microbiota of mice was analyzed after treating WT and ALDH2 mice with high-fat diets. Observed amplicon sequence variants (ASVs) and Shannon indices showed that there were no differences between ALDH-M, ALDH-F, WT-M, and WT-F (Figure 4A,B) and indicated that ALDH deficiency might not affect the α-diversity of gut microbiota. Principal coordinate analysis (PCoA) revealed a distinct clustering of the microbiota composition for each group. ALDH-M had a distinct microbiota composition that clustered differently from those of the three other groups (Figure 4C), which indicated that the ALDH2 gene mutation might affect β-diversity in males. Figure 5 shows that ALDH2-M exhibited decreased abundance of the *Actinobacteria* phylum compared to in WT-M. Sixfold higher abundances of the *Deferribacteres* phylum were observed in ALDH2-M compared to in WT-M (Figure 5A and Table 3). The decreased abundance of the *Coriobacteriia* and *Bacilli* classes, and higher abundances of the *Deferribacteres* class were observed in ALDH2-M compared to in WT-M mice. Additionally, the ALDH2-M group revealed a decreased *Bacilli* class compared to that in the ALDH2-F group (Figure 5B and Table 3). At the order level, a decreased abundance of *Lactobacillales*, *Coriobacteriales*, and *Bacillales,* and higher abundance of *Deferribacteres* were observed in ALDH2-M compared to in WT-M. ALDH2-M had decreased abundance of *Lactobacillales* compared to that in ALDH2-F (Figure 5C and Table 3). The differential enrichment of specific bacteria is shown in both cladograms and histograms based on an LDA score of > 2 for pairwise comparisons. LEfSe analysis indicated that WT-F had an increased proportion of *Streptococcaceae* compared to WT-M (Figure 5D). ALDH2-M mice exhibited an increased abundance of *Deferribacteraceae* and *Mucispirillum,* and decreased abundance of *Lactobacillaceae* compared to those of WT-M mice (Figure 5E). Furthermore, ALDH2-F mice exhibited an increased proportion of *Marinifilaceae* and *Odoribacter* compared to those in ALDH2-M (Figure 5F).

## 4. Discussion

In the present study, the ALDH2 mutation exacerbated HFD-induced NAFLD, including increased body weight, fat accumulation, liver injury, and insulin resistance in male mice. These changes were not observed in female mice carrying the same ALDH2 mutation. More severe insulin resistance, an elevated AUC of glucose, and increased white adipose tissue weight were seen in the ALDH2-M compared with those in WT-M, but no significant difference was found between female WT and ALDH2 mice. Decreased liver PGC-1α disrupts insulin signaling associated with insulin resistance in NAFLD [24]. Kotani et al. reported that GLUT4 glucose transporter deficiency increases hepatic lipid production and peripheral lipid utilization [25]. Transgenic mice lacking or overexpressing GLUT4 respectively decrease or increase whole-body insulin sensitivity [26]. In our study, the gene expressions of IRS2, PGC-1α, and GLUT4 were significantly decreased in ALDH2-M compared with those in WT-M. OGTT and HOMA-IR indices show that glucose tolerance and IR were more severe in the ALDH2-M group than those in the WT-M group. Female WT or ALDH2 mice did not exhibit a similar phenotype. The prevalence of NAFLD was reported to be 18.8–30.2% in the US, in Europe around 24%, and in Asia around 23.3–31.9% [27,28]. The prevalence of NAFLD in Japan was reported to be 29.7%, with a threefold difference in the mean prevalence between men (41.0%) and women (17.7%) [29]. Furthermore, Kishimoto et al. reported that, after the injection of β-estradiol, the primary female reproductive hormone, there was marked activated hepatic total ALDH in male rats. In contrast, the total ALDH activity was decreased in females following treatment with the primary male hormone, testosterone [30]. Louet et al. also reported that estrogen may be protective against the development of insulin resistance and diabetes [31]. Based on these findings, the ALDH2 gene may influence visceral fat deposition, causing insulin resistance in males.

Gut-microbiota dysbiosis affects endogenous alcohol production through the fermentation of carbohydrates in the intestinal lumen. Endogenous alcohol contributes by inducing liver damage and aggravating the pathology of NAFLD [32]. Obese mice that had not been fed any alcohol exhaled a higher alcohol amount than lean mice did [33]. Clinical studies also demonstrated that patients with NAFLD presented higher blood ethanol concentrations than healthy patients [34], suggesting that the endogenous ethanol production contributes to worsening liver damage [32,33,34]. The HFD leads to gut-microbiota dysbiosis [32,34]; our study used ALDH2-mutated mice to explore HFD-induced NAFLD and found that ALDH2 mutation resulted in more severely damaged liver function than in WT mice, but this result was only found in male and not female mice. The endogenous estrogens play a protective role in NASH, which may explain why the prevalence of NAFLD decreases in females [19].

The gut-microbiota composition and diversity of male WT and ALDH2 mice were affected by being fed an HFD for 16 weeks, but this was not observed in the female mice. These results demonstrated that potential changes of gut microbiota may be associated with the defective ALDH2 exacerbation of HFD-induced NAFLD. The gut microbiota affects fat accumulation, insulin resistance, and liver functions, and causes inflammation [35]. Jiao et al. reported that the gut microbiome may contribute to insulin resistance [35], and Farnesoid X receptor (FXR) deficiency enriched *Deferribacteraceae*, which positively correlated with ALT and hepatic triglyceride levels [36]. In our study, ALDH2-M presented a more relative abundance of *Deferribacteraceae* compared to in WT-M and WT-F. ALDH2-M also exhibited the highest levels of serum ALT and the most hepatic triglyceride content compared to other groups. Peng et al. indicated that FXR deficiency resulted in decreased ALDH expression [37]. These results suggested a potential relationship among FXR, ALDH, and *Deferribacteraceae*, which needs to be further examined. The administration of probiotics, *Lactobacillus rhamnosus*, *Lactobacillus acidophilus*, and *Lactobacillus plantarum,* which belong to the *Lactobacillales* order, reportedly reduced HOMA-IR and improved OGTT values [38,39]. Our findings showed that ALDH2-M had a decreased relative abundance of *Lactobacillales* compared to that in other groups. ALDH2-M also exhibited elevated HOMR-IR and insulin resistance. These results suggest that the decreased relative abundance of *Lactobacillales* may contribute to insulin resistance. Furthermore, a previous study indicated that the relative abundance of *Odoribacter* positively correlated with the Matsuda index, which indicated that *Odoribacter* may ameliorate insulin resistance [40]. These results support our findings of decreased *Odoribacter* in ALDH2-M compared to in ALDH2-F. Taken together, increased *Deferribacteraceae* and decreased *Lactobacillales* and *Odoribacter* may contribute to differential responses to HFD in ALDH-deficient male and female mice.

## 5. Conclusions

In our study, the ALDH2 gene mutation may have exacerbated HFD-induced NAFLD and affected gut-microbiota composition and diversity in male WT and ALDH2 mice, but not in female mice. The high prevalence of ALDH2 mutation may be one of the causes of NAFLD in East Asia. Our findings implicate the ALDH2 gene mutation as an important component in the pathogenesis of NAFLD. However, sex hormones may also be one of the effect factors. For future work, we plan to perform related studies to verify that estrogen can activate hepatic ALDH2 in male mice and may be protective against the development of insulin resistance and NAFLD.

## Figures and Tables

**Table 1:** Real-time PCR primers used in this study.

Gene (GenBank ID)	Orientation	Sequence (5′-3′)	UPL
IRS2 (NM_001082121.1)	Forward Reverse	gtccaggcactggagcttt gcgcttcactctttcacga	53
GLUT4 (NM_009204.2)	Forward Reverse	gacggacactccatctgttg gccacgatggagacatagc	5
PGC-1α (NM_008904.2)	Forward Reverse	cccatacacaaccgcagtc gaacccttggggtcatttg	6
β-actin (NM_007393.3)	Forward Reverse	ctaaggccaaccgtgaaaag accagaggcatacagggaca	64

**Table 2:** Basic physiological value, liver injury index, and concentration of hepatic triglycerides of WT and ALDH2 mice fed an HFD.

Characteristic	Male		Female		*p* Values for Two-Way ANOVA		
	WT	ALDH2	WT	ALDH2	Main Effect of Gender	Main Effect of Genotype	Interaction (G × G’)
Diet intake (g/mouse/day) Terminal body weight (g)	2.74 ± 0.14 38.2 ± 3.6	2.79 ± 0.14 44.5 ± 4.8 ^#^	2.48 ± 0.04 27.2 ± 3.6	2.51 ± 0.07 26.6 ± 3.8	<0.001 <0.0001	0.1381 0.0985	0.7928 0.471
White adipose tissue weight (g)	3.16 ± 1.02	4.86 ± 0.66 ^#^	2.05 ± 1.07	1.37 ± 0.93	<0.0001	0.203	0.007
ALT (U/L)	61.3 ± 26.2	142.9 ± 57.9	82.3 ± 37.9	92.9 ± 50.3	0.4772	0.0338	0.0939
AST (U/L)	261.1 ± 104.0	346.3 ± 97.6	263.6 ± 119.2	248.4 ± 90.7	0.2697	0.4152	0.0939
Hepatic TG (mg/dl)	920.2 ± 151.5	1143.1 ± 154.6	887.7 ± 162.8	871.3 ± 95.9	0.0195	0.0997	0.0594

WT: wild type; ALDH2: ALDH2 knock-in mice; HFD: high-fat diet; ANOVA: two-way analysis of variance; G: gender; G’: genotype. Values are presented as mean ± standard deviation (SD) for *n* = 5–6 mice in each group; ^#^ *p* < 0.05 compared with WT-M group. TG: triglycerides.

**Table 3:** Relative abundance of gut microbiota by meta-analysis.

Taxonomy	WT-M	ALDH-M	WT-F	ALDH-F
Actinobacteria (phylum)	2.896 ± 0.016	0.780 ± 0.002 *	1.167 ± 0.004	1.331 ± 0.008
*Coriobacteriia* (class)	2.254 ± 0.013	0.586 ± 0.002 *	0.715 ± 0.002	1.049 ± 0.008
*Coriobacteriales* (order)	2.254 ± 0.013	0.586 ± 0.002 *	0.715 ± 0.002	1.049 ± 0.008
Deferribacteres (phylum)	0.836 ± 0.012	5.096 ± 0.028 *	0.125 ± 0.001	1.715 ± 0.017
*Deferribacteres* (class)	0.836 ± 0.012	5.096 ± 0.028 *	0.125 ± 0.001 ^#^	1.715 ± 0.017
*Deferribacterales* (order)	0.836 ± 0.012	5.096 ± 0.028 *	0.125 ± 0.001 ^#^	1.715 ± 0.017
Firmicutes (phylum)	40.521 ± 0.122	37.079 ± 0.114	45.770 ± 0.029	41.753 ± 0.071
*Bacilli* (class)	6.712 ± 0.018	2.299 ± 0.018 *	10.101 ± 0.042 ^#^	7.380 ± 0.023 ^#^
*Lactobacillales* (order)	5.497 ± 0.016	1.733 ± 0.015 *	9.055 ± 0.046 ^#^	6.591 ± 0.022 ^#^
*Bacillales* (order)	1.215 ± 0.004	0.566 ± 0.003 *	1.046 ± 0.006	0.789 ± 0.003
Proteobacteria (phylum)	5.032 ± 0.025	6.786 ± 0.036	3.929 ± 0.005	7.333 ± 0.033
*Deltaproteobacteria* (class)	3.182 ± 0.025	5.858 ± 0.038	1.861 ± 0.006	5.831 ± 0.037
*Desulfovibronales* (order)	3.182 ± 0.025	5.858 ± 0.038	1.856 ± 0.006 ^#^	5.831 ± 0.037

Relative abundance of taxa listed under study groups are percentages. Data expressed as mean ± SD. * *p* < 0.05 compared to WT-M, ^#^ *p* < 0.05 compared to ALDH2-M.

## Data Availability

The data presented in this study are available in the article.
